# Beta Glucan: Health Benefits in Obesity and Metabolic Syndrome

**DOI:** 10.1155/2012/851362

**Published:** 2011-12-11

**Authors:** D. El Khoury, C. Cuda, B. L. Luhovyy, G. H. Anderson

**Affiliations:** Department of Nutritional Sciences, Faculty of Medicine, University of Toronto, Toronto, ON, Canada M5S 3E2

## Abstract

Despite the lack of international agreement regarding the definition and classification of fiber, there is established evidence on the role of dietary fibers in obesity and metabolic syndrome. Beta glucan (*β*-glucan) is a soluble fiber readily available from oat and barley grains that has been gaining interest due to its multiple functional and bioactive properties. Its beneficial role in insulin resistance, dyslipidemia, hypertension, and obesity is being continuously documented. The fermentability of *β*-glucans and their ability to form highly viscous solutions in the human gut may constitute the basis of their health benefits. Consequently, the applicability of *β*-glucan as a food ingredient is being widely considered with the dual purposes of increasing the fiber content of food products and enhancing their health properties. Therefore, this paper explores the role of *β*-glucans in the prevention and treatment of characteristics of the metabolic syndrome, their underlying mechanisms of action, and their potential in food applications.

## 1. Introduction

Obesity has reached global epidemic proportions with more than one billion adults affected by this chronic disorder [1]. Coronary artery disease, stroke, insulin resistance, type 2 diabetes, hypertension, and metabolic syndrome are well-known medical comorbidities associated with excess body weight [2]. The metabolic syndrome is defined by a combination of three or more of the following: (a) abdominal circumference >102 cm (40′′) for men and 88 cm (35′′) for women, (b) hypertension, (c) hyperglycemia, and (d) dyslipidemia (elevated triacylglyceride concentrations and low levels of high-density lipoproteins (HDL) in blood) [3]. It is directly associated with increased risk of type 2 diabetes and cardiovascular diseases.

Many studies have examined the potential of diets and dietary components as a first-line intervention in the prevention and treatment of metabolic syndrome [4]. Accordingly, various dietary constituents, foods, and dietary practices, capable of controlling blood glucose, insulin and lipids, blood pressure, and food intake have been identified. Although the ideal dietary pattern for patients with metabolic syndrome has not been defined, there is growing evidence that high intakes of fruits, vegetables, legumes, and cereals are beneficial [5–11]. Many of their benefits have been attributed to their low-glycemic properties and their dietary fiber content. However, dietary fibers in fruits, vegetables, legumes, and cereals are poorly defined and vary greatly in characteristics.

The focus of this review is on beta glucan (*β*-glucan), which is a dietary fiber readily found in oat and barley bran. *β*-glucan is a relatively inexpensive milling byproduct, and it is added to foods on the assumption that this will contribute to health benefits. *β*-glucans are predominantly found in the internal aleurone and subaleurone cell walls [12–14]. The content of *β*-glucan varies with environmental conditions during endosperm development and is regulated by (1 → 3,1 → 4)-*β*-glucan endohydrolase (EC 3.2.1.73 also known as licheninase or 1,3-1,4-beta glucanase) to facilitate endosperm cell-wall degradation during germination [15]. Among cereals, the highest content (g per 100 g dry weight) of *β*-glucan has been reported for barley: 2–20 g (65% is water-soluble fraction) and for oats: 3–8 g (82% is water-soluble fraction). Other cereals also contain *β*-glucan but in much lower amounts: sorghum 1.1–6.2 g, rye 1.3–2.7 g, maize 0.8–1.7 g, triticale 0.3–1.2 g, wheat 0.5–1.0 g, durum wheat 0.5-0.6 g, and rice 0.13 g [16]. Other sources of *β*-glucan include some types of seaweed [17] and various species of mushrooms such as Reishi, Shiitake, and Maitake [18].

Canada is a major producer of both oats and barley, producing 2297.6 and 7605.3 thousand metric tonnes of oats and barley, respectively, in 2010/2011 [19, 20]. In 2007, Canada was the 5th leading producer of barley and the 2nd leading producer of oats worldwide [21]. Fractions rich in *β*-glucans are readily obtained from cereal grains by dry milling followed by sieving and air classification processes or by wet milling followed by sieving and solvent extractions [22]. These approaches result in concentrates or isolates containing 8–30% and 95% *β*-glucans, respectively. During oat processing, oat bran and aleurone layers can be milled from oat groat, creating the bran as a major byproduct. Oat *β*-glucan is found in greater concentrations in the bran as compared to the whole-oat groat and commercial oat bran contains 7–10% *β*-glucan [23]. However, extraction of pure *β*-glucan isolates is not straightforward and relatively costly since the aleurone and subaleurone cell walls also enclose starch, protein, and lipids [24]. Thus, pure *β*-glucan isolates are often ignored in food product development and relatively inexpensive oat and barley bran or flour fractions are typically used.

The objective of the current review is to illustrate the role of *β*-glucan, as a soluble and fermentable fiber, in the prevention and treatment of various metabolic syndrome-linked diseases. *β*-glucan is then compared to other soluble and fermentable dietary fibers, clarifying whether the effects of *β*-glucan on health and disease are unique. An overview of definitions and types of fiber is provided first and then followed by an in-depth examination of the health benefits associated with *β*-glucan, its mechanisms of action, and its potential food applications.

## 2. Dietary Fibers: Characteristics, Definitions, Classifications, and Analytical Methods

Scientific and regulatory bodies around the world define fiber differently. The challenge of defining fiber is probably best exemplified by the 10-year process that was required to achieve an international legal definition for dietary fiber by Codex [25]. Most definitions of fiber address its biological, chemical, and nutritional characteristics while recent regulatory requirements have created the need for analytical definitions. Fibers can also be categorized based on their physical and chemical properties as well as their physiological effects. The following sections outline some characteristics of fiber, its various definitions and classifications as well as the analytical approaches used for its quantification. Prior to an in-depth examination of *β*-glucan, a brief overview describing the role of dietary fibers in metabolic syndrome will be given.

### 2.1. Characteristics of Dietary Fibers

Four categories of fiber definitions have been identified [26], each of which addresses a different characteristic of fiber. In general, these categories describe fiber based on its source, chemical composition, digestibility, metabolic fate, and physiological effects. Depending on which characteristic is used to define fiber, various carbohydrates can be included under the definition. Each category of definitions has its advantages and disadvantages and because of the variation in fiber types, a combination of different approaches is usually necessary in order to define fiber in a comprehensive manner.

Biological definitions describe the origins of fiber and have historically referred to nonstarch polysaccharides from plant cell walls. The earliest formal definition of fiber refers to the source of fiber: “Dietary fibre is the proportion of food which is derived from the cellular walls of plants, which is digested very poorly in human beings” [27]. This definition was soon updated to include nondigestible polysaccharides that are not part of the plant cell wall [28], in order to account for storage carbohydrates such as guar gum. However, this definition remains limiting as fibers can also be obtained from animal, fungal, bacterial, and synthetic sources. Categorization based on source is also complicated by the inability of analytical methods to distinguish fiber origin [29].

Chemically, fiber can be described based on its chain length and type of linkages between each monomeric unit. This provides a very precise and unequivocal meaning; however, deciding on the appropriate chain length for fiber has been difficult. The Codex definition for fiber indicates that fibers have a degree of polymerization (DP) ≥ 10, but also includes a footnote that the decision on whether to include carbohydrates with a DP > 2 (i.e., oligosaccharides) is up to national authorities [30]. Fibers can also be described based on the chemical bonds between their monomeric units as nonstarch polysaccharides are typically linked by *β*-linkages; however, this specification would exclude resistant starches, which contain *α*-1,4 linkages.

The physiological effects of fiber refer to its nondigestibility and metabolic effects. Nondigestibility in the small intestine is fundamental to fiber and was part of the first definition put forth by Trowell [27]. However, nondigestibility and a lack of absorption by the small intestine alone do not guarantee favourable physiological effects. Depending on physicochemical properties, fibers have a range of physiological consequences including viscosity in the upper gastrointestinal tract [31, 32], fermentation in the colon [33], and prebiotic effects [34, 35]. These effects in the gastrointestinal tract improve laxation and increase stool bulking and also have metabolic consequences including improvements in serum lipids and postprandial glycemia and promotion of satiety.

Analytical definitions are used for labelling and inspection purposes. They often describe an “official method,” which is simple and reproducible enough to minimize dispute. The risk with these types of definitions is that they are not able to recognize new fiber compounds, which may have significant and beneficial health implications. Consequentially, the “official method” has to be continually updated to measure these new compounds. This type of definition is very practical from a regulatory point of view; however, it alone does not actually describe any characteristics of fiber and an analytical method should only be part of a formal regulatory definition.

### 2.2. Definitions of Dietary Fibers

The most recent definitions for fiber generally address at least one of four characteristics: (1) source, (2) chemical characteristics, (3) resistance to digestion, and (4) beneficial biological effects. With the advances of food science, isolation, modification, and synthesis of many fibers are possible, which have resulted in some jurisdictions distinguishing between naturally occurring fibers from plant source and isolated or synthesized fibers. Others have chosen not to adopt this division by either considering all nondigestible carbohydrates as fiber or only those carbohydrates that are intrinsic and intact in plants.  Table 1 lists examples of such definitions based on this division.

### 2.3. Classification of Dietary Fibers

As seen in the previous section, fibers are often classified by their source (plant, animal, isolated, synthetic, etc.), but they can also be classified according to chemical, physical, or physiological criteria [36, 37].

#### 2.3.1. Chemical (Polymer Length and Types of Linkages)

Chemical classification can divide carbohydrates based on their chain length, or DP: sugars (DP 1-2), oligosaccharides (DP 3–9), and polysaccharides (DP ≥ 10). Oligosaccharides are either (a) maltodextrins (*α*-glucans), principally resulting from the hydrolysis of starch, or (b) non-*α*-glucan such as raffinose and stachyose, fructo- and galactooligosaccharides and other oligosaccharides. Polysaccharides may be divided into starch (*α*-1,4 and 1,6 glucans) and nonstarch polysaccharides, which primarily consist of plant cell wall polysaccharides such as cellulose, hemicelluloses, and pectin but also includes plant gums, mucilages, and hydrocolloids. However, some carbohydrates do not fit into this categorization. For instance, inulin may have from 2 to 200 fructose units and thus can be both oligo- and polysaccharide [35].

#### 2.3.2. Physical (Solubility and Viscosity)

Fibers are most commonly characterized based on their solubility. Distinction between soluble and insoluble dietary fibers is based on the solubility characteristics of dietary fiber in hot aqueous buffer solutions [38]. Solubility of dietary fiber structure cannot be simply described as the solubility in water. Solubility of dietary fibers is rather defined as dissolved or liquefied in a buffer and enzyme solution modeled after, but not necessarily identical to, the aqueous enzyme solutions or slurries present in the human system [39]. Insoluble fibers primarily consist of cellulose and some hemicelluloses, resistant starch, and chitin while soluble fibers include pectins, *β*-glucans, galactomannan gums, mucilages, and some hemicelluloses. Solubility can be used as a means to broadly characterize the physiological effects of fibers. In general, insoluble fibers increase fecal bulk and the excretion of bile acids and decrease intestinal transit time (i.e., laxative effect). Soluble fibers increase total transit time by delaying gastric emptying and also slow glucose absorption [40]. Although this characterization of fiber is used to generalize the effects of each fiber type, only soluble viscous fibers delay gastric emptying time and slow glucose absorption while nonviscous soluble fibers primarily act as a substrate for microbial fermentation in the colon [33].

#### 2.3.3. Physiological (Rate of Digestion and Fermentation)

The rate at which a carbohydrate is digested is determined by a number of factors, including the rate at which carbohydrate leaves the stomach and becomes available for absorption as well as diffusion of released sugars occurs from food bolus [41]. Thus, the rate at which carbohydrates leave the food matrix and the ability for amylase to act on the carbohydrate is an important determinant of glucose absorption rate and resulting blood glucose levels. Based on digestion, carbohydrates can be categorized as rapidly or slowly digested or even resistant. Resistant carbohydrates include plant cell wall polysaccharides, gums, fructans, resistant maltodextrins, and resistant starches.

These carbohydrates that resist digestion make their way to the large intestine, where they may be fermented by the gut microflora [33] or have prebiotic effects [34]. However, not all fiber is fermented. Short-chained fatty acids produced from fermentation are mainly sourced from resistant starches [42, 43]. Insoluble fibers (e.g., lignins, cellulose, and some hemicelluloses) are resistant to fermentation while soluble fibers (e.g., pectins, gums, mucilages, and some hemicelluloses) are more completely fermented by colonic microflora [33]. A prebiotic is a nondigestible food ingredient that selectively stimulates the growth and/or activity of a limited number of colonic bacteria and subsequently improves host health [44]. Prebiotic fibers alter the balance of the gut microflora towards what is considered to be a healthier one [34] and includes fructans and resistant starches [45].

### 2.4. Analytical Methods for Fiber Quantification

For food labelling purposes, it is important that analytical methods complement the fiber definition in a given jurisdiction. Fibers are typically measured by enzymatic-gravimetric methods, although there are also gravimetric, nonenzymatic-gravimetric, and enzymatic chemical methods. High-performance liquid chromatography (HPLC), gas liquid chromatography (GLC), and ion-exchange chromatography are also used [29]. Fibers recovered with enzymatic-gravimetric methods include cellulose, hemicelluloses, pectins, some other nonstarch polysaccharides, lignin and some resistant starch. Soluble and insoluble fibers can also be measured separately by this method [46]. However, these methods do not capture inulin and polydextrose and partially measure resistant starch. To remedy this, separate procedures have been proposed to quantify these other compounds. For instance, *β*-glucans can be measured by AOAC method 995.16, AAC method 32-23, and a method by McCleary and Codd [47]. Resistant starch, oligofructan, inulin, fructo-oligosaccharides, and polydextrose can also be measured independently by several methods [29].

However, these methods incompletely measure all fibers included in the Codex definition, and the use of some or all of these methods could result in underestimation of some fibers as well as overestimation of others due to double counting. The McCleary method [48] (AOAC 2009.01) was proposed to accompany the Codex definition as it allows for measurement of a complete range of dietary fiber components, including nondigestible oligosaccharides and resistant starches, in one test, without double counting or missing fiber compounds [48]. This method uses extended enzymatic digestion at 37°C, followed by gravimetric isolation and quantitation of high-molecular weight dietary fiber and liquid chromatography to quantitate low-molecular weight dietary fibers [49]. It is also particularly important for food labelling that fiber analysis be completed on foods as they would be eaten in order to provide more accurate fiber values that account for the effects of processing and cooking procedures [49].

For analysis of *β*-glucan, two AOAC methods have been adopted in oats, barley, and their products. Both methods are enzymatic colorimetric methods that use lichenase to cleave 1,3 *β*-bonds in *β*-glucan to produce oligosaccharides of various lengths that are subsequently hydrolyzed to glucose with amyloglucosidase, and then the glucose is assayed colorimetrically [39]. The AOAC method 992.28 is applicable to measure 1–12% *β*-glucans in oat and barley fractions, unsweetened oat cereals, and ready-to-eat cereals [50]. The AOAC method 995.16 is used to analyze *β*-glucan content in flours from whole grains, milling fractions, and unsweetened cereal products [47]. In addition to AOAC methods, there are other methods including enzyme-linked immunosorbent assay (ELISA) [51], near-infrared spectroscopy [52], and fluorescence assay of complex formed between *β*-glucan and calcofluor [53], which are all specifically designed to measure *β*-glucan.

### 2.5. Dietary Fibers in the Prevention and Treatment of Metabolic Syndrome

Dietary fibers have been strongly implicated in the prevention and treatment of various characteristics of the metabolic syndrome. The beneficial effect of fiber-rich foods and isolated fibers, both insoluble and soluble, on obesity, cardiovascular diseases, and type 2 diabetes has been shown in randomized studies [6, 11]. Diets rich in fiber improve glycemic control in type 2 diabetes [54], reduce low-density lipoprotein (LDL) cholesterol in hypercholesterolemia [55–57], and contribute positively to long-term weight management [58]. In epidemiological studies, positive associations were noted between increased cereal consumption, a source of both insoluble and soluble fibers, and reduced risk of metabolic syndrome, cardiovascular diseases, and markers of systemic inflammation [59–61]. Diets rich in whole-grain foods have also been negatively associated with metabolic syndrome [6, 8, 11].

In comparison to insoluble fibers, soluble fibers are more potent in attenuating the presence of components of the metabolic syndrome in both animals and humans. Addition of *α*-cyclodextrin, a soluble dietary fiber, to high-fat-diet-fed male Wistar rats for 6 weeks attenuated weight gain and increases in plasma cholesterol and triglyceride levels while also preventing increased fecal fat content relatively to the control high fat diet [62]. Serum leptin levels were normalized and insulin sensitivity index was improved. A diet supplemented with the soluble fibers from *Plantago Ovata* husks (psyllium) and methylcellulose over 10 weeks improved obesity and lipid profile and ameliorated the unbalanced secretions of the inflammatory tumor necrosis factor-*α* (TNF-*α*) and adiponectin by the visceral adipose tissue in obese Zucker rats [63]. The diet supplemented with the soluble fermentable fiber* Plantago Ovata* husks also resulted in the greatest improvement in hyperinsulinemia and hyperleptinemia, and lowered the production and accumulation of lipids in the liver. This effect was associated with activation of the AMP-activated protein kinase (AMPK) system [63], known to increase fatty acid oxidation and decrease fatty acid synthesis [64]. In humans, a daily intake of at least 5 g of soluble fiber, particularly from whole-grain foods and fruits, reduced the presence of metabolic syndrome in patients with type 2 diabetes by 54% [65]. Moreover, a high fiber meal, in which refined-wheat flour was replaced with whole-wheat flour (16.8 g/meal), increased postprandial adiponectin concentrations in diabetic females [66]. In a cross-sectional study on diabetic men, adiponectin levels were 19% higher in the highest quintile of cereal fiber intake than in the lowest quintile [67]. High adiponectin levels are associated with improved glycemic control and insulin sensitivity, a more favorable lipid profile and reduced inflammation in diabetic females [68].

Among soluble fibers, *β*-glucan is the most frequently consumed and is associated with reduced presence of insulin resistance, dyslipidemia, hypertension, and obesity. The role of *β*-glucan in the prevention and treatment of these determinants is discussed in the following sections.

## 3. Beta Glucan, Obesity, and Metabolic Syndrome

Increased interest in *β*-glucan in the last two decades arises from its functional and bioactive properties. Of all fibers, its health benefits have been the most extensively documented, and the use of health claims with *β*-glucan-containing foods has been allowed in several countries including Canada, the United States of American, Sweden, Finland, and the United Kingdom [69]. Moreover, no human adverse effects have been reported following the consumption of a diet rich in *β*-glucan from oat or barley flour or their extracts [70].

### 3.1. Definition of Beta Glucan

Glucans are glucose polymers, classified according to their interchain linkage as being either *α*- or *β*-linked [71]. *β*-glucans are a heterogeneous group of nonstarch polysaccharides, consisting of D-glucose monomers linked by *β*-glycosidic bonds [72]. The macromolecular structure of *β*-glucan depends on both the source and method of isolation. The simplest glucan is the linear and unbranched *β*-(1,3)-D-glucan, found among prokaryotes and eukaryotes [73]. Another simple structural type occurs mostly in the nonlignified cell walls of cereal grains, and consist of linear *β*-(1,3;1,4)-D-glucans [74]. Glucans from barley, oats, or wheat are found in cell walls of the endosperm, while being concentrated in the aleurone layer of barley, oats, wheat, sorghum, and other cereals. Branched structures of *β*-glucans consist of *β*-(1,3)- or *β*-(1,4)-glucan backbone with either (1,2)- or (1,6)-linked *β*-glucopyranosyl side branches [71]. They are major structural components of the cell walls of yeast, fungi, and some bacteria [75]. The side branched *β*-(1,3;1,2)-D-glucan is only present in the type 37 capsule of the bacterium *Streptococcus pneumonia* [73]. Branched *β*-(1,4;1,6)-D-glucan and *β*-(1,3;1,6)-D-glucan are found in different groups of yeast, fungi, and algae [71]. In algae, *β*-glucans are present as storage polysaccharides or cell wall components. Some cyclic (1,2) and (1,3;1,6) *β*-glucans were also isolated from various bacteria. These glucans are important for plant-microbe interactions, and act as signalling molecules during plant infection [76]. Besides differences in type of linkage and branching, *β*-glucans can vary in terms of frequency and length of branching, degree of branching, molecular weight (from 10^2^ to 10^6^ daltons), polymer charge, and/or solution conformation (random coil or triple or single helix) as well as solubility [77]. All these factors play a role in shaping *β*-glucan-associated biological activities, and should be taken into consideration by researchers when discussing the physiological impacts of *β*-glucans.

The *β* linkages in the polymer render *β*-glucan nondigestible [78]. Moreover, *β* glucans are highly fermentable in the caecum and colon [79]. In comparison to other oat fractions, *β*-glucan induced the maximum growth rate and cell proliferation rate of bacteria isolated from human intestine and the maximum lactic acid productions [80]. The solubility of *β*-glucans is highly influenced by their structures [81]. However, no sharp distinction exists between the insoluble and soluble fractions and the ratio is highly dependent on the extraction conditions of the soluble fiber [82]. The (1 → 3)-*β*-glucans with a high degree of polymerization (DP > 100) are completely insoluble in water [83]. This conformation allows for stronger interactions and associations between chains than between the chains and water molecules. Solubility increases as the degree of polymerization is lowered. The composition of the side substituted branches and the frequency of these branches also determine the solubility of *β*-glucan molecules [84]. A single (1 → 6)-*β* linked glucose side can transform the glucan into a more soluble form in comparison to its unbranched molecule [85]. Most studies have examined the structure and properties of water-soluble *β*-glucans, in contrast to water-insoluble ones [86, 87].

Depending on physicochemical characteristics, various biological functions of *β*-glucans have been described. This review elaborates on the role of *β*-glucans in the prevention and treatment of the metabolic syndrome; however, a description of the immunomodulatory functions of *β*-glucans will be briefly examined in the following section.

### 3.2. Beta Glucan and Immunomodulation

Among polysaccharides that act as immunostimulants, *β*-glucans were found to be the most effective against infectious diseases and cancer [88]. The immunological potency of *β*-glucans varies with the molecular mass, solution conformation, backbone structure, degree of branching as well as the cell type that is targeted [89].

The role of 1,3 *β*-glucans from yeast, fungi, mushrooms, and seaweed as biological immunomodulators has been well documented in the past 40 years [90]. *In vitro*, animal and human studies have shown that 1,3 *β*-glucans can enhance the responsiveness and function of immune cells, stimulating both humoral and cellular immunity [91]. *In vitro* studies demonstrated that *β*-glucans can enhance the functional activity of macrophages and activate the anti-microbial activity of mononuclear cells and neutrophils [72, 92]. *In vivo* studies of a variety of *β*-glucans on the responses to pathogen infections in animals have observed increased microbial clearance and reduced mortality in lethally infected animals when exposed to *β*-glucans [93, 94]. Very few human studies examined the immune function of *β*-glucans. Three clinical studies demonstrated that pretreatment of high-risk surgical patients with intravenous yeast *β*-(1,3; 1,6)-D-glucan decreased the infection incidence, shortened intensive care unit length stay, and improved survival in comparison to a saline placebo injection [95–97].

### 3.3. Beta Glucan and Parameters of the Metabolic Syndrome

There is growing interest in the understanding of the association between *β*-glucans and determinants of the metabolic syndrome. Most studies have used plant *β*-glucans as functional viscous dietary fibers in the management of various components of the metabolic syndrome. Only two studies described a protective role of nonplant *β*-glucans in metabolic syndrome. In obese hypercholesterolemic men, consumption of 12 g of yeast *β*-(1,3;1,6)-D-glucan over 8 weeks lowered total cholesterol concentrations, and increased HDL-cholesterol levels only 4 weeks after discontinuation of glucan intake [98]. One study completed in mice found that effects of chronic consumption of chitin-glucan from a fungal source improved metabolic abnormalities induced by a high fat diet [99]. Chitin-glucan is a cell wall polysaccharide-based three-dimensional network in which the central core contains branched chitin-*β*-1,3 glucan. In this particular study, chitin-glucan decreased high fat diet-induced body weight gain, fat mass development, fasting hyperglycemia, glucose intolerance, hepatic triglyceride accumulation, and hypercholesterolemia, irrespective of caloric intake. These beneficial effects were mainly attributed to restoration of the composition and/or activity of gut bacteria.

The ability of plant *β*-glucans, which will be referred to as “*β*-glucans” in the following sections, to form highly viscous solutions in the human gut is thought to be the basis of its health benefits. These benefits include lowering postprandial glucose and insulin responses, decreasing cholesterol levels, and potentiating the feelings of satiety. Beta glucan has the ability to form highly viscous solutions because it is a linear, unbranched, nonstarchy polysaccharide composed of *β* (1–4) and *β* (1–3)-linked glucose molecules [100]. However, the viscosity of *β*-glucan depends on the molecular weight, solubility, and concentration [100–102]. For instance, high molecular weight *β*-glucans produce a higher viscosity than *β*-glucans with low molecular weights. Whether the ability to form highly viscous solutions at low concentrations provides *β*-glucan with unique health benefits in comparison to other soluble and fermentable dietary fibers has received little investigation. The role of *β*-glucan compared to other soluble fibers in affecting the components of the metabolic syndrome will be discussed in the following sections. 

#### 3.3.1. Beta Glucan and Insulin Resistance

Insulin resistance, whether or not accompanied with hyperglycemia, and type 2 diabetes are well-established components of metabolic syndrome [103].

Several soluble fibers, including *β*-glucan, psyllium and guar gum, reduce postprandial glucose and insulin responses, and improve insulin sensitivity both in diabetic and nondiabetic individuals [104–110]. In healthy individuals, a beverage containing 25 g/200 mL each of resistant dextrins or soluble corn fiber, a class of soluble fibers isolated from wheat or corn, attenuated postprandial glycemic, and insulinemic responses relatively to a control glucose solution (25 g glucose/200 mL of the test beverage) [111]. Arabinoxylan consumption, at 15 g/day over 6 weeks, significantly lowered the postprandial responses of serum glucose and insulin to a liquid meal challenge test in overweight subjects with impaired glucose tolerance [112]. In stroke-prone spontaneously hypertensive rats, psyllium supplementation (5%) prevented insulin resistance in response to a high-caloric diet given from 5 to 9 weeks of age [113].

Beta glucan also contributes to glycemic control. Several factors were found to influence such an interaction, including dose, food form, and molecular weight. Dose of *β*-glucan is important in the regulation of the effects of this fiber on glycemic responses. Relative to other fibers, smaller amounts of *β*-glucan are required to bring about reductions in postprandial glucose and insulin responses in healthy subjects [114, 115], type 2 diabetic patients [116, 117] and moderately hypercholesterolemic men and women [118]. In subjects with noninsulin-dependent diabetes mellitus, consumption of three breakfasts with 4, 6, and 8.6 g of oat *β*-glucan in a breakfast cereal significantly decreased the peak and average increases in glucose and insulin as compared to the control [116]. A significant relationship between the amount of *β*-glucan in cereals and plasma glucose peak or area under the glucose curve was also observed. Similarly, a linear dose-dependent decrease in glycemic responses was noted in response to breads containing varied doses of barley *β*-glucan ranging from 0.1% to 6.3% [119]. Consumption of oat bran providing 7.3 g  *β*-glucan in a breakfast cereal or 6.2 g in a bar lowered postprandial glucose responses more than an oat bran breakfast cereal providing 3.7 g *β*-glucan in type 2 diabetic subjects [120]. The consumption of oat bran flour containing 9.4 g of *β*-glucan lowered postprandial glycemia in type 2 diabetic patients in comparison to a glucose load [117]. In addition, oat bran crisps containing 3 g of *β*-glucan also reduced postprandial glycemia, although the reduction was only half as large as the effect induced by oat bran flour containing 9.4 g *β*-glucan. In hypercholesterolemic individuals, the addition of 5 g of oat *β*-glucan per day to a beverage consumed for 5 weeks attenuated both glucose and insulin responses compared to the control beverage [121]. However, in healthy individuals, larger doses of *β*-glucan are needed in order to alter their glycemic homeostasis. Unlike diabetic subjects [117], a 3 g oat *β*-glucan dose did not affect postprandial glycemic response in healthy subjects [122] while the intake of muesli with 4 g oat *β*-glucan lowered postprandial blood glucose responses in comparison to a reference meal without muesli and *β*-glucan in healthy individuals [122, 123].

Food form has also an influence on *β*-glucan's regulation of glycemia. Incorporating a high dose of oat bran *β*-glucan (5.2 g) into fettucini did not significantly lower postprandial blood glucose relative to the fettucini alone in healthy subjects [124]. This is perhaps because wheat pasta itself has a low glycemic response. Molecular weight is another determinant of viscosity in addition to the concentration [101], and modulates the influence of *β*-glucan on glycemia. A drink containing 5 g of oat *β*-glucan with a molecular weight 70 000 Da significantly lowered postprandial glucose and insulin levels relative to a rice drink control, while a similar drink containing barley *β*-glucan of molecular weight 40 000 Da had no effect [121]. 

Reduced insulin responses have consistently been observed following the ingestion of *β*-glucan [122, 125–127]. As in the case of glycemia, dose is an important factor in shaping insulin responses to *β*-glucan. A consistent decrease in insulin secretions was dose-dependently observed in overweight individuals in response to oat *β*-glucan, with significant changes reported at a dose of at least 3.8 g of *β*-glucan [127]. Some studies have found the impact of *β*-glucan on insulinemia to be independent of its glycemic effect. In healthy men, barley-enriched pasta, containing 5 g of *β*-glucan, induced a significant reduction in insulinemia in comparison to the control pasta without any apparent effect on glycemia [128]. Similarly, in healthy subjects, the ingestion of 50 g rye bread, containing 5.4 g of *β*-glucan, reduced postprandial insulinemic responses without a parallel reduction in glucose responses as compared with the control bread [109]. It was hypothesized that the low glycemic indices of pasta and rye bread may attenuate the effects of *β*-glucan on glucose responses.

Several mechanisms have been suggested to explain the glucose- and insulin-lowering effects of soluble fibers, more precisely *β*-glucan. One of the mechanisms includes the ability of soluble fibers to form viscous solutions. Delayed gastric emptying occurs with increased digesta viscosity [129–131], slowing subsequent digestion and absorption [132]. High digesta viscosity decreases enzyme diffusion [133] and stimulates the formation of the unstirred water layer [134], decreasing glucose transport to enterocytes [31]. Reducing the viscosity of guar gum following acid hydrolysis resulted in concurrent loss of its clinical efficacy [31]. A relationship was noted between guar gum viscosity and its glycemic response. Moreover, it was stated that the viscosity of *β*-glucan could account for 79–96% of the changes in glucose and insulin responses to 50 g glucose in a drink model [135].

Evidence for delayed stomach emptying following the consumption of *β*-glucan emerged from human and animal studies. The quantity of exogenous glucose appearing in plasma was 18% lower, during the first 120 min, following the polenta meal with 5 g oat *β*-glucan in comparison to the control polenta meal in overweight individuals [136]. Similarly, the addition of ^13^C-labelled glucose to a meal containing 8.9 g *β*-glucan, consumed over 3 days, lowered the appearance of exogenous ^13^C-glucose in plasma by 21% relatively to a control meal without *β*-glucan [137].

Short-chain fatty acids resulting from the anaerobic bacterial fermentation of soluble dietary fibers such as *β*-glucan in the colon [138] offer another explanatory mechanism for the protective effects of soluble fibers on glucose and insulin homeostasis. The short-chain fatty acids propionic and butyric acid increased muscle expression of the insulin-responsive glucose transporter type 4 (GLUT-4) via the peroxisome proliferator-activated receptor (PPAR) *γ* [113]. The activation of PPAR*γ* also increased GLUT-4 content in adipocytes [139]. Stroke-prone spontaneously hypertensive rats consuming psyllium supplementation, at 5% in a high caloric diet, witnessed improved muscle insulin sensitivity via short-chain fatty acid-induced increased membrane GLUT-4 expression in comparison to cellulose supplementation [113].

In conclusion, due to its viscosity and fermentability, *β*-glucan plays a significant protective role against insulin resistance in various populations.

#### 3.3.2. Beta Glucan and Dyslipidemia

Individuals with metabolic syndrome often present with atherogenic dyslipidemia, characterized by elevated concentrations of triacylglycerols and low levels of HDL cholesterol in blood [3]. This lipid profile presents an individual with a high risk for cardiovascular disease.

Soluble fibers have the most reported beneficial effects on cholesterol metabolism. In a meta-analysis, soluble fibers pectin, psyllium, oat bran, and guar gum were all proven to be equally effective in reducing plasma total and LDL cholesterol levels [55]. When included within a low saturated fat and cholesterol diet, soluble fibers lowered LDL cholesterol concentrations by 5–10% in hypercholesterolemic and diabetic patients [55, 108]. The consumption of 14 g per day of *Plantago Ovata *husk for 8 weeks induced a significant reduction in total cholesterol, LDL cholesterol, and oxidized LDL in mild-moderate hypercholesterolemic patients [140]. Conversely, soluble fibers from barley, oats, psyllium, and pectin had no effect on HDL cholesterol levels [55, 141].

Variable effects of soluble fibers on triglyceridemia have been noted. In two meta-analyses, soluble fibers, including barley, oats, psyllium, and pectin, had no significant impacts on triglyceride concentrations [141]. Other studies have described hypotriglyceridemic effects of soluble fibers in various populations. In a study on type 2 diabetic patients, the intake of a high-soluble fiber diet (25 g/day) over a period of 6 weeks lowered triglyceride concentrations by 10.2% [142]. The soluble fiber in *Plantago Ovata* husk reduced triglyceridemia in human secondary cardiovascular disease risk trials, when consumed at 10.5 g/day over 8 weeks [143]. Similarly, the consumption of arabinoxylan at 15 g/day over 6 weeks significantly reduced postprandial triglyceride responses in overweight subjects with impaired glucose tolerance [112]. Discrepancies in findings could be attributed to the variability in fiber structure, the degree of solubility and viscosity, different administered doses, the duration of administration, and baseline triglyceride levels of the subjects.

The effect of *β*-glucan on lipid parameters has been intensively investigated; however, differing results have been found. These inconsistencies in findings may be explained by several factors including the sources, dose and molecular size of *β*-glucans, dietary composition, food preparation, food state (solid versus liquid), subject's baseline cholesterol concentrations, and study design [144] as well as the cultivar of barley and oat being used and their growing conditions [145, 146]. Although varied effects of barley and oat-derived *β*-glucans have been reported on lipid homeostasis, they were not established as significant differences since *β*-glucan content of these two cereals is almost comparable [147, 148]. In the following sections, the impacts of barley and oat *β*-glucans on lipid parameters will be separately discussed.

A limited effect of barley *β*-glucan on lipid parameters has been described and the dose of barley *β*-glucan appears to be a major determinant of this effect. In a meta-analysis of randomized clinical trials, the consumption of 3 to 10 g of barley *β*-glucan per day, over 4 to 6 weeks, significantly lowered total and LDL cholesterol in subjects with different dietary backgrounds [141]. In another meta-analysis of 8 randomized controlled trials, participants receiving 3 to 10 g of barley *β*-glucan per day, over a duration ranging between 4 and 12 weeks, had significant reductions in total cholesterol, LDL cholesterol, and triglycerides in comparison to control group participants, irrespective of whether a low-fat or a Step I diet was given [144]. Moreover, the consumption of pearl barley, providing 7 g of *β*-glucan per day over 12 weeks, significantly reduced serum concentrations of total cholesterol and LDL cholesterol in hypercholesterolemic Japanese men [149]. Both total and LDL cholesterol concentrations were significantly reduced following the consumption of the high barley *β*-glucan diet (6 g/day), in comparison with the diet low in barley *β*-glucan (0–0.4 g/day) in hypercholesterolemic subjects [150, 151]. In contrast, daily ingestion of 10 g of barley *β*-glucan over 4 weeks in the form of bread, cakes, muffins or savory dishes, had no effect on serum lipoprotein profile in hypercholesterolemic men in comparison with the control group [152]. In addition, neither 5 g nor 10 g of barley *β*-glucan consumed daily in a beverage over 5 weeks had a significant impact on serum lipids in hypercholesterolemic subjects as compared with control [121]. Thus, in addition to dose, the food vehicle delivering barley *β*-glucan also affects its regulation of lipid responses.

Despite conflicting results, oat *β*-glucans were found to be strongly effective in modulating plasma lipid parameters. As in the case of barley *β*-glucan, the ingested dose of oat *β*-glucan appears as a limiting factor. The US Food and Drug Administration and Health Canada have accepted 3 g as an effective daily intake of oat *β*-glucan to reduce serum LDL cholesterol [74, 153]. In a meta-analysis on oats containing 2 to 10 g per day of *β*-glucan, a net change of −3.1 mg/dL to −15.5 mg/dL for total cholesterol and of −2.9 mg/dL to −14.3 mg/dL for LDL cholesterol was observed [55]. A significantly greater serum cholesterol reduction was reported after the intake of 4 g of *β*-glucan as compared to 2 g from oat bran or oat meal incorporated into muffins, cereals, and shakes [154]. Increasing the dose to 6 g of *β*-glucan did not provide any further reduction in serum cholesterol concentrations. Similarly, a beverage providing 5 g of *β*-glucan per day from oats significantly lowered total and LDL cholesterol over a period of 5 weeks compared to a control beverage, in hypercholesterolemic individuals [121]. No additional benefit was reported on serum lipids when increasing the daily dose of oat *β*-glucan to 10 g. A bread containing 6 g of oat-derived *β*-glucan significantly improved HDL cholesterol and diminished LDL cholesterol, non-HDL cholesterol, total cholesterol/HDL cholesterol ratio, and LDL cholesterol/HDL cholesterol ratio, over 8 weeks compared to whole-wheat bread, in overweight individuals with mild hypercholesterolemia [155]. Similarly, the consumption of 6 g/day of concentrated oat *β*-glucan in the form of powder for 6 weeks significantly reduced both total and LDL cholesterol in hypercholesterolemic adults, with the reduction in LDL cholesterol being greater than that in the control group [156]. A once-daily consumption of 4 g of *β*-glucans from oats, incorporated into a ready-meal soup, reduced LDL cholesterol levels by 3.7% over 5 weeks in a group of hyperlipidemic healthy subjects as compared with a control diet [157]. In contrast, in some studies, the reductions were small and nonsignificant, around less than 5% for LDL cholesterol, in comparison to control groups [158–162]. Food vehicle, rather than dose, seems to explain such minimal lipid responses to oat *β*-glucan ingestion in these studies. A once-daily consumption of 20 g of an oat bran concentrate (containing 3 g of oat *β*-glucan) in the form of cereal for 12 weeks did not affect total cholesterol and LDL cholesterol as compared to 20 g wheat bran (control) [161], nor did 4 weeks of 5.9 g of oat bran *β*-glucan administered daily in bread and cookies [162].

The mode of administration of *β*-glucan is another determinant to consider when explaining such variability in results since structural changes in *β*-glucan may result from food processing or storage of barley and oat products. The consumption of oat *β*-glucan in a variety of foods, such as muffins and cereals, effectively lowered LDL cholesterol [163], suggesting that the structure and molecular weight of oat *β*-glucan are maintained in these products. On the other hand, the effects of oat *β*-glucan administered in bread are controversial. The consumption of bread providing 140 g of rolled oats per day led to an 11% reduction in serum total cholesterol concentrations [164]. However, other studies found no hypocholesterolemic effect of incorporating oats into bread [158, 165–167]. Bread making can cause significant depolymerization of *β*-glucan, primarily induced by *β*-glucanase enzymes present in wheat flour [162, 168]. The activation of these enzymes depends on the processing technique used in bread making.

The varied responses of cholesterol-rich lipoproteins to *β*-glucans could be also attributed to differences in molecular weight and solubility of the fibers. Molecular weight, solubility, and viscosity are important physicochemical properties of *β*-glucan, which are strongly affected by the genetic attributes of oat and barley grains [169]. For instance, oat *β*-glucans have a higher molecular weight than barley *β*-glucans [102, 170–172]. Only 15–20% of barley *β*-glucans are water soluble while almost 70% of the oat *β*-glucans are soluble in water [173]. Relatively to barley *β*-glucan, the higher molecular weight of oat *β*-glucan is attributed to a greater content and frequency of side branches rather than to a higher degree of polymerization, explaining its higher degree of water solubility [83, 85]. As viscosity is highly influenced by the molecular weight and solubility of *β*-glucan, a lower molecular weight and/or solubility of *β*-glucan are expected to reduce its resultant viscosity and consequently its cholesterol-lowering effects. Highly water-soluble *β*-glucan, with moderate to high molecular weight, reduced serum LDL cholesterol better than *β*-glucan with low water-solubility and low molecular weight [174]. This explains the lower reported effects of barley *β*-glucan on lipid parameters as compared to oat *β*-glucan.

The hypocholesterolemic properties of *β*-glucans are explained by various mechanisms some of which are shared with other soluble dietary fibers. Altering bile acid excretion and the composition of bile acid pool is one of the mechanisms. Dietary fibers are associated with increased bile acid excretion and increased activity of cholesterol 7*α*-hydrolase, a major enzyme leading to cholesterol elimination in the body [175]. Beta glucans can decrease the reabsorption of bile acids and increase their transport towards the large intestine [176], promoting their increased microbial conversion to metabolites and their higher excretion, subsequently inducing increased hepatic synthesis of bile acids from circulating cholesterol [177]. This mechanism is strongly related to *β*-glucan-induced increased viscosity in the small intestine [128, 178, 179] and consequently slowed gastric emptying, digestion, and absorption [179]. In addition, some soluble fibers decrease the absorption of dietary cholesterol by altering the composition of the bile acid pool. In fact, oat bran increased the portion of total bile acid pool that was deoxycholic acid [180], a microbial byproduct of bile acid which decreases the absorption of exogenous cholesterol in humans [181].

The fermentation of some soluble fibers, including *β*-glucan, provides another explanation for their cholesterol-lowering effects. Fermentation changes the concentration of bile acids in the intestinal tract of rats [177] as well as the production of short-chain fatty acids, which influence lipid metabolism. For example, propionate is thought to suppress cholesterol synthesis, though results are still inconclusive [182–186] and acetate may contribute to the lowering of cholesterol circulating levels [187]. It should be well noted that differences between soluble fibers in the relative production of acetate, propionate, butyrate, and total short-chain fatty acids do exist. Oat *β*-glucan ferments more rapidly than guar gum, reflected in higher concentrations of total short-chain fatty acids, in general, and of acetate and butyrate, in particular [32]. However, such differences may not be that important to generate varied degrees of hypocholesterolemic impacts among soluble fibers.

Few mechanisms, most not clearly elucidated, have been suggested in order to explain the hypotriglyceridemic properties of soluble fibers, including *β*-glucan. Two mechanisms include a possible delay in the absorption of triglycerides in the small intestine [188], as well as a reduced rate of glucose absorption [189]. Glucose-induced hypertriglyceridemia, via the process of *de novo* lipogenesis, is well established in the literature [190]. Furthermore, direct inhibition of lipogenesis by soluble fibers is also suggested as an explanatory mechanism. The hypotriglyceridemic effect of oligofructose was reported to result from the inhibition of hepatic lipogenesis via the modulation of fatty acid synthase activity [191, 192]. In an *in vitro *study, *β*-glucan extracts from oat and barley flour inhibited the *in vitro* intestinal uptake of long-chain fatty acids and cholesterol and downregulated various genes involved in lipogenesis and lipid transport in rats [147].

In conclusion, *β*-glucan possesses similar hypocholesterolemic properties as other soluble dietary fibers. However, the hypotriglyceridemic impacts of *β*-glucan have not been fully determined and warrant further investigation. Additionally, further studies need to be conducted in order to optimize *β*-glucan's hypolipidemic dose and to investigate the long-term effect of *β*-glucan supplementation on blood lipid chemistry. The eventual goal would be to combine *β*-glucan supplementation with other dietary means of controlling blood lipids, and to consequently prevent the need for cholesterol-lowering drugs in hyperlipidemic patients.

#### 3.3.3. Beta Glucan and Blood Pressure

Hypertension is another core component of the metabolic syndrome, and is an established risk factor for heart diseases, stroke, and renal diseases [193].

The effects of soluble dietary fibers, including *β*-glucan, on arterial blood pressure have been the least studied among the components of the metabolic syndrome. In one meta-analysis, increased dietary fiber consumption provided a safe and acceptable means to reduce blood pressure in patients with hypertension [194]. In a randomized crossover study on hyperlipidemic adults, small reductions in blood pressure were reported following the intake of a high-fiber diet containing *β*-glucan or psyllium (8 g/day more than the unsupplemented food in the control diet) over 4 weeks [195]. In another randomized parallel-group study on hypertensive and hyperinsulinemic men and women, the oat cereal group (standardized to 5.52 g/day of *β*-glucan) experienced a significant reduction in systolic and diastolic blood pressure in comparison to the low-fiber cereal control group (<1 g/day of total fiber) over 6 weeks [196]. Similarly, in a randomized double-blind placebo-controlled trial on participants with untreated elevated blood pressure or stage 1 hypertension, the consumption of 8 g/day of supplemented soluble fiber from oat bran over 12 weeks significantly reduced both systolic and diastolic blood pressure in comparison to the control [197].

Various mechanisms underlying the antihypertensive effects of soluble dietary fibers have been hypothesized. Insulin resistance is a major underlying mechanism contributing to the development of hypertension [198] and soluble fibers may affect blood pressure by modulating insulin metabolism [199]. Reductions in plasma cholesterol, observed following the ingestion of soluble fibers, are also associated with improvements in endothelium-mediated vasodilation [200, 201]. Preliminary findings in animals support a direct relationship between changes in circulating cholesterol levels and blood pressure [202]. Finally, soluble fiber-induced weight loss, which will be discussed in the coming section, has also been suggested as a potential mechanism. Increased body weight is a strong risk factor for hypertension [203]. 

In conclusion, additional studies are still needed in order to fully elucidate the mechanisms underlying the protective effects of soluble fibers against hypertension. Moreover, the association between *β*-glucan and blood pressure remains to be further explored.

#### 3.3.4. Beta Glucan, Satiety, and Obesity

Central obesity is a well-established component of the metabolic syndrome [3]. One potential countermeasure to the current obesity epidemic is to identify and recommend foods that spontaneously reduce energy intake by inducing satiation and increasing satiety.

Dietary fiber has documented effects on satiety, food intake, and body weight although the outcomes have not been consistent [204]. A number of randomized controlled trials have shown weight reduction with diets rich in dietary fiber or dietary fiber supplements [205–208], while others have not [209]. However, a meta-analysis of 22 clinical trials concluded that a 12 g increase in daily fiber intake is associated with a 10% reduction in energy intake and a 1.9 kg reduction in weight during an average study duration of 3.8 months [204]. More specifically, the soluble dietary fiber glucomannan, which has a strong water-holding capacity, resulted in a significantly greater reduction of weight, when consumed at a dose of 1.24 g daily for 5 weeks in conjunction with an energy-restricted diet, as compared to the placebo energy-restricted group [210].

Despite the clear association between soluble fibers and weight loss, their effects on subjective measures of satiety are not conclusive. However, soluble fibers with viscosity-producing properties, including guar gum, pectin, psyllium, and *β*-glucan, are more strongly associated with reduced hunger and/or appetite perceptions than low/no fiber condition [211]. For example, the addition of 2.5 g of guar gum to a semisolid meal prevented an increase in appetite, hunger, and desire to eat in overweight male volunteers [212]. The soluble resistant dextrins promoted, in a dose-dependent manner, increased satiety when added to desserts and to carbohydrate-based meals [213–215]. Moreover, a nutrition bar containing guar gum (5.7 g guar gum and 9.1 g other fibers) increased perceived fullness and decreased hunger sensations as compared to a reference bar (6.4 g dietary fiber) [216].

Barley, a source of *β*-glucan, possesses satiating properties when fed intact. Subjects described to be significantly less hungry before lunch after consuming barley—but not wheat—and rice-containing foods [217]. Barley-based foods enhanced as well satiety when compared to a high-glycemic index food or a food with no dietary fiber [218–220]. This effect does not appear specific to one type of barley, as different cultivars of barley produced an equivalently greater satiety feeling, up to 180 min postprandially, in comparison to white wheat bread [218].

In contrast to whole barley, both positive [128, 221–223] and negative [220, 224–226] effects of *β*-glucan on satiety have been described. A beverage containing oat *β*-glucan, at levels of 10.5 g/400 g portion and 2.5 g and 5 g/300 g portion, increased fullness sensation in comparison to the beverage free of fiber in healthy volunteers [222, 227]. Similarly, a preload of 5.2% barley *β*-glucan-enriched biscuits significantly suppressed appetite ratings in healthy adolescents, without modifying subsequent food intake at lunch, as compared with control biscuits [228]. In healthy participants, a 3% barley *β*-glucan-enriched bread induced a higher reduction of hunger and increase in fullness and satiety as compared to the control bread. This was also associated with a significant reduction of energy intake at the subsequent lunch [223]. In contrast, a meal replacement bar containing 1.2 g of barley *β*-glucan (from 8.0 g barley), consumed at breakfast on 2 consecutive days by healthy subjects, did not modify appetite scores or energy intake at subsequent lunch in comparison to a control bar containing only 0.3 g *β*-glucan (from 6.8 g oats) [226]. Moreover, muesli containing 4 g of oat *β*-glucan did not induce differential satiating effects than an isocaloric portion of cornflakes in healthy individuals [123], as a dose of 2 g of *β*-glucan in cereal test meals did not affect satiety ratings in comparison to isocaloric glucose load in overweight participants [225].

The efficacy of *β*-glucan on satiety depends on several factors. Dose is one of the major determinants. A beverage (300 g) containing 5 g of oat dietary fiber (2.5 g of *β*-glucan) produced significantly higher ratings of satiety than the fiber-free beverage [227]. However, when the dose was raised to 10 g of oat fiber (5 g of *β*-glucan), no additional impact on satiety scores was reported [227]. The physical effects of *β*-glucans on the ingesta appear to be fundamentally important in shaping their satiating properties. This effect is largely determined by molecular size and solubility of *β*-glucans [229]. The molecular weight of *β*-glucan, a major determinant of solubility, varies from 31 to 3100 kilodaltons [230] and can change during isolation, purification, and extraction procedures [231]. Such variability in the molecular weight and solubility of *β*-glucan may explain its varied impacts on satiety. Finally, the carrier food also plays a role in defining the interaction of *β*-glucans with satiety. Almost all studies that did not report any significant influence of *β*-glucan on satiety used solid or semisolid foods as carrier foods, unlike studies that incorporated *β*-glucan into liquid meals [227]. Solid foods are known to increase satiety and decrease hunger more effectively than liquid ones [232]. Thus, the larger satiating effect of solid food per se may mask the satiating potential of *β*-glucans.

Since almost all studies did not account for these factors and were run under different experimental conditions (different *β*-glucan dose, various molecular weights and food sources of the fiber, different dosing protocols, and diverse types of subjects), ranking the satiating power of *β*-glucan is still not possible at this stage. Moreover, another concern to be addressed in future studies is the type of control to use. No dietary fiber that may function as a control for satiety studies has been actually identified. In almost all studies, the control food was the same food with either a lower amount or a complete absence of *β*-glucans.

As the effect of *β*-glucan on satiety is still unclear, its effect on body weight regulation is less clear. In a study on diabetic patients, the supplementation of *β*-glucan from oats, at a dose of 9 g/day over 24 weeks, did not have any significant effect on body weight [69, 233]. In another study on hyperlipidemic patients, weight differences were not observed following the consumption of a diet rich in oat *β*-glucan (8 g/day), over 1 month, as compared to the control group [195]. It should be noted that the body weight was not the primary concern of these studies as they focused on changes in blood sugar or blood lipids. Even at moderate (5-6 g/d) and high (8-9 g/d) doses, the addition of oat *β*-glucan to an energy-restricted diet did not enhance the effect of energy restriction on weight loss in overweight women after a period of 3 months [234]. In contrast, hypercholesterolemic Japanese men consuming a mixture of rice and pearl barley with a high *β*-glucan content (7 g/day), for 12 weeks, experienced a significant reduction in body mass index, waist circumference, and visceral fat in comparison to the placebo group consuming rice alone [149]. Variations in the food sources of *β*-glucan, rather than in the dose and the duration of administration, may explain such contradictions in findings and appear as critical determinants of body weight regulation.

The satiating properties of soluble dietary fibers have been explained by various mechanisms, all of which are related to several stages in the process of appetite regulation such as taste, gastric emptying, absorption, and fermentation [235]. Firstly, the viscosity of soluble fibers plays an important role in their ability to induce satiety [222, 236, 237]. The most viscous *β*-glucan-enriched beverage increased perceived satiety significantly more than the beverage containing the same amount of fiber but with enzymatically lowered viscosity [227]. A higher viscosity meal delays gastric emptying [130, 131, 238] and slows the digestion and absorption of nutrients, more precisely glucose, due to reduced enzymatic activity and mucosal absorption [31, 239], leading to early satiety sensations. The overall gastric emptying rate of healthy volunteers, as assessed by the paracetamol absorption test, was slower after the high viscosity oat bran-enriched beverage as compared to the low viscosity drink [240]. Secondly, the lower palatability of fiber-rich meals may affect food intake in a negative manner [241–243]. A strong inverse relationship is described between palatability and satiation [244]. When chronically consumed, products enriched with *β*-glucan had lower sensory acceptance [121, 245]. Third, the reduced glycemic and insulinemic responses to soluble fibers, including *β*-glucan, can be also responsible for their satiating properties. A significant inverse relationship is reported between satiety and glucose and insulin responses to carbohydrate-rich breakfast cereals [246, 247] and to beverages with different glycemic effects [248]. However, other studies did not report any association of glucose and insulin postprandial levels with satiety [249, 250]. They suggested that the release of putative satiety peptides is a more crucial component of mechanisms initiating and maintaining satiety. Such statement leads to the fourth suggested mechanism that delineates the role of short-chain fatty acids in appetite control. Short-chain fatty acids regulate the release of various gut hormones, which play an important role in satiety signaling. Most *β*-glucan consumed is fermented in the caecum and colon, producing short-chain fatty acids [79]. The role of short-chain fatty acids in appetite regulation and the potential underlying mechanisms will be elucidated in the following sections.

**(i) Short-Chain Fatty Acids and Appetite Regulation**

Dietary fibers pass as unaffected through the small intestine, and upon reaching the colon, anaerobic bacteria degrade some dietary fibers via a fermentation process, yielding short-chain fatty acids. The fermentability of soluble fibers by colonic microbiota is greater than that of insoluble fibers. Pectin, resistant starches, gums, and polyfructans (such as inulin) are the most highly fermented substrates. Around 80% of short-chain fatty acids present in the human colonic lumen are in the form of acetate, propionate, and butyrate [251]. About 90% of these short-chain fatty acids are rapidly absorbed in the colon; butyrate is almost entirely used by the colonocytes as their preferred energy substrates [252] while propionate is primarily removed by the liver [251]. On the other hand, acetate passes more freely into the peripheral circulation [253]. Several functions are attributed to short-chain fatty acids, being recently proposed as key energy homeostasis signaling molecules [254].

Accumulating evidence has attributed the satiating effects of fermentable carbohydrates to short-chain fatty acids, their major fermentation products [255]. Short-chain fatty acids regulate appetite through several mechanisms. First, short-chain fatty acids have a role in slowing gastrointestinal motility, thus controlling digestion and nutrient absorption and eliciting an anorexigenic effect. The majority of the studies linking short-chain fatty acids to gastrointestinal motility stems from ruminant animal studies [256], where the production of short-chain fatty acids is greater than that in humans due to differences in gut physiology [257]. However, there are some studies on nonruminants showing that short-chain fatty acids may regulate the overall transit time of the digesta through the large intestine [258, 259]. Such responses were hypothesized to occur via three possible pathways: (1) short-chain fatty acid stimulation of the vagal nerves in the gut, (2) a direct effect of short-chain fatty acids on intestinal smooth muscle tone, and (3) as a consequence of the indirect changes in the secretion of peptide YY (PYY) and other regulatory peptides known to play a role in gastrointestinal motility [260]. In addition, short-chain fatty acids were suggested to regulate gastrointestinal motility by affecting the release of the gastrointestinal 5-hydroxytryptamine (5-HT) via the activation of the free fatty acid receptor 2 (FFA2), the major receptor for short-chain fatty acids. 5-HT or serotonin is a neurotransmitter in the central nervous system, known to modulate mood, behavior, and appetite [261]. Though the central actions of 5-HT are the most documented, 95% of endogenous 5-HT is found peripherally in the gastrointestinal tract [262]. The activation of various 5-HT receptor subtypes stimulates vagal nodose neurons and consequently prolongs colonic transit time [263, 264]. Short-chain fatty acids also regulate appetite by modulating the release of various appetite-related hormones throughout the gastrointestinal tract [265]. The effects of short-chain fatty acids on the release of some of these gut hormones, including PYY, glucagon-like peptide 1 (GLP-1), cholecystokinin (CCK), and ghrelin, will be discussed in the following sections, providing partial explanations for the reported impacts of soluble dietary fibers in general, and of *β*-glucan specifically, on satiety hormones and consequently on appetite and food intake.

**Peptide YY**

Peptide YY is a 36-amino acid peptide, first isolated from porcine upper small intestine [266]. Two circulating forms of PYY are released by L cells in the distal gut, PYY_1–36_ and PYY_3–36_, which is the truncated major circulating form [267]. PYY is secreted throughout the entire length of the gastrointestinal tract, with the highest concentrations found in the colon and rectum [268]. Circulating PYY levels are the lowest in the fasting state and increase following the consumption of a meal, peaking at 1-2 hours and remaining elevated for several hours. Peripheral PYY administration decreased food intake and body weight gain in rats [269]. Similarly, it decreased appetite and food intake both in lean and obese humans [269, 270].

An increased PYY response was consistently described following the consumption of various soluble dietary fibers. Postprandial PYY clearly increased after the consumption of psyllium-enriched test meals in healthy volunteers [271]. The consumption of PolyGlycopleX, a novel functional fiber complex manufactured from three dietary fibers to form a highly viscous polysaccharide with high water-holding and gel-forming properties, for 3 weeks resulted in significantly increased fasting PYY levels as compared to the control product in healthy adults [272]. Moreover, a meal tolerance test in overweight and obese adults consuming 21 g of oligofructose for 3 months resulted in a greater increase in PYY concentrations as compared to the placebo group, concomitant with a reduced self-reported caloric intake [273].

The ability of *β*-glucan to increase PYY release was reported in various population groups. In healthy subjects, bread enriched with 3 g barley *β*-glucans induced a 16% higher overall PYY response in comparison to the control bread [223]. Even in overweight men and women, PYY levels responded positively and in a dose-responsive manner to increasing oat *β*-glucan concentrations, ranging from 2.16 g to 5.45 g per serving, in the first 4 hours after a meal [274].

The fermentation process of *β*-glucan and the subsequent generation of short-chain fatty acids provide a major explanatory mechanism for *β*-glucan-induced PYY release. The direct infusion of short-chain fatty acids into rabbit and rat colons significantly increased PYY secretions [275, 276]. The stimulatory effects of short-chain fatty acids on PYY secretions are mainly attributed to a direct interaction between short-chain fatty acids and PYY cells. In fact, FFA2 (also known as GPR43), the major receptor for short-chain fatty acids, is colocalized with PYY immunoreactive enteroendocrine L cells both in rat ileum and human colon [259, 277].

**Glucagon-Like Peptide 1**

Glucagon-like peptide 1 is cosecreted with PYY from the intestinal L cells, encoded by the proglucagon gene [278]. It is described with a potent incretin effect, stimulating insulin secretion in a glucose-dependent manner. Circulating GLP-1 levels rise following nutrient ingestion, in proportion to the energetic content of the meal [279]. An acute intracerebroventricular administration of GLP-1 to rodents induced a decline in short-term energy intake [280], and was associated with a reduced body weight following repeated administration [281]. Similarly, an intravenous infusion of GLP-1 both in normal weight and in obese subjects resulted in a dose-dependent reduction in food intake [282].

The effects of *β*-glucan on GLP-1 release have not been yet elucidated; however, the effects of other soluble fibers have been investigated. Variable GLP-1 responses to soluble dietary fiber intake were described, whether elevated, inhibited, or unaffected. The exposure to a diet supplemented with 10% oligofructose for 4 weeks increased the number of GLP-1-producing L-cells as well as endogenous GLP-1 production in the proximal colon of male Wistar rats in comparison to a standard diet [283]. In humans, a standard breakfast containing galactose (50 g) and guar gum (2.5 g) increased, extendedly, GLP-1 release in healthy women as compared with a standard control breakfast [284]. In contrast, in normal-weight males, resistant (pregelatinized) starch (50 g) produced a smaller GLP-1 response than digestible starch (50 g) [285]. On the other hand, the ingestion of pasta enriched with a small amount of psyllium fiber (1.7 g) did not modify postprandial GLP-1 responses in comparison to the control pasta in healthy subjects [286]. Such discrepancies in findings could be attributed to differences in the structures and food sources of ingested soluble fibers and their administered doses.

Colonic fermentation appears to be essential in explaining GLP-1 release in response to soluble dietary fibers, despite inconsistent findings. Though supplementation with fermentable carbohydrates has been consistently associated with increased colonic proglucagon mRNA expression [287–293], only few studies detected increased plasma GLP-1 circulating levels in parallel [288–290, 293–295]. Rats fed high doses of the fermentable inulin-type fructans (100 g/day), over 3 weeks, had higher mRNA expressions in the proximal colon and plasma concentrations of GLP-1 as compared to those fed a standard diet [288]. The exposure of male Wistar rats to a diet supplemented with 10% of inulin-type fructans, for 3 weeks, resulted in a higher caecal pool of GLP-1, an increase in GLP-1 and of its precursor proglucagon mRNA concentrations in the proximal colon, and an increase in the circulating levels of GLP-1 as compared to the standard diet [289]. In normal-weight adults, the microbial fermentation of 16 g of soluble fructan per day, over 2 weeks, induced increased levels of GLP-1 in circulation as compared to the control dextrin maltose [296]. A strong association between postprandial hydrogen production and plasma GLP-1 concentrations was also reported. On the contrary, others have shown no effect of fermentable carbohydrates on circulating GLP-1 levels, whether acutely [297] or over a short duration of 6 days [298]. Based on these findings, the duration of supplementation is an important factor to consider when suggesting fermentation as a basis for soluble fibers-induced GLP-1 release. A sufficient time of 2-3 weeks must be given in order to allow adaptation of the gut microbiota to the additional fermentable carbohydrate within the diet for maximal fermentation to take place [299] and for GLP-1 levels in circulation to be subsequently affected.

**Cholecystokinin**

Cholecystokinin was among the first hormones shown to modulate food intake [300]. It is secreted from the I cells of the small intestine in response to food ingestion [301]. Cholecystokinin circulating levels rise rapidly after a meal, reaching a peak within 15 minutes. It was found to reduce food intake when infused both in rodents and humans [301, 302]. In fact, plasma CCK levels are strongly associated with subjective measurements of satiety in women [303].

Limited studies described the interaction between soluble dietary fibers and CCK release. Various soluble fibers, including hydrolyzed guar gum (20 g) in obese females [304], *β*-glucan in barley pasta (15.7 g) in healthy men [128], and isolated fibers from oatmeal and oat bran (8.6 g) in healthy men [305], produced greater and longer-lasting postprandial CCK levels in comparison to low-fiber or placebo meals. A study on overweight women revealed a dose-dependent effect of increased oat *β*-glucan concentrations, ranging from 2.16 to 5.68 g per serving, on CCK levels in the first 4 hours after a meal, with a significant CCK release observed at a minimum dose of 3.8 g of *β*-glucan [127].

The role of fermentation and more specifically short-chain fatty acids in regulating CCK release is still poorly understood. In pigs, ileal infusion of short-chain fatty acids did not affect CCK circulating levels [306]. Thus, the fermentation process per se does not explain CCK responses to *β*-glucan ingestion. Additional mechanisms underlying the stimulatory effects of *β*-glucan on CCK secretions remain to be explored.

**Ghrelin**

Ghrelin is the only known orexigenic hormone in the gut. It was initially identified as an endogenous ligand for growth hormone secretagogue receptor (GH-SR) in rat stomach [307]. Circulating ghrelin levels increase before meals and fall rapidly after eating [308]. Both central and peripheral administration of ghrelin increased food intake and body weight in rodents [309, 310].

The effects of soluble fibers, including *β*-glucan, on postprandial ghrelin are not fully understood. The consumption of a small amount (4 g) of noncaloric soluble psyllium fiber with water suppressed postprandial ghrelin levels as effectively as a 585-Kcal mixed meal in healthy women [311]. On the other hand, postprandial plasma ghrelin did not decrease following gastric distention with a noncaloric liquid meal containing 21 g of soluble guar gum fiber in comparison to carbohydrate-, protein-, and fat-rich meals [312]. Moreover, a 300-Kcal meal enriched with 23 g of psyllium fiber inhibited postprandial suppression of plasma ghrelin levels [313]. When compared to a control breakfast, a soluble arabinoxylan fiber-enriched breakfast (6 g) induced a shorter postprandial ghrelin decline [314] whereas bread enriched with 3 g barley *β*-glucans resulted in 23% lower ghrelin responses than a control bread [223]. Discrepancies in findings could be explained by variations in the physical and chemical properties of ingested soluble fibers, their different administered doses, and the forms of ghrelin being measured in circulation.

Several mechanisms were suggested to explain fiber-induced ghrelin suppression, most importantly fermentation. Feeding a diet supplemented with 10% of the fermentable inulin to rats over 3 weeks significantly reduced ghrelin levels in comparison to a standard diet [289]. The ingestion of 56 g of high-fructose corn syrup (HFCS) plus 24 g inulin induced greater postprandial ghrelin suppression as compared to HFCS without inulin, both at 4.5 and 6 hours, in healthy subjects [315]. Such colonic fermentation may reduce ghrelin via increasing circulating PYY levels. Administration of PYY to humans reduced serum ghrelin levels [316]. In addition to colonic fermentation, other mechanisms were also hypothesized. A possible inner-gastric pathway may operate through gastric somatostatin, which is released following the consumption of beet fiber in diabetic individuals [317]. Somatostatin administration decreased ghrelin secretion in rats [318] and lowered circulating ghrelin levels in humans [319]. In addition, GLP-1 release in response to soluble fibers is another potential mechanism. Infusion of GLP-1 into isolated rat stomach suppressed ghrelin secretions [320].

In conclusion, there is evidence for the satiety efficacy of *β*-glucan. Such satiating capacity appears to be comparable to that of other soluble viscous and fermentable fibers. Although several mechanisms may explain the satiating properties of *β*-glucan, the generation of short-chain fatty acids through colonic fermentation has the most documented effects. Short-chain fatty acids affect satiety by primarily modulating the release of various appetite-regulating hormones, including PYY, GLP-1, and ghrelin. However, other yet unknown mechanisms, independent of short-chain fatty acids, may be involved in the regulation of gut hormones by *β*-glucans. Since research in this area is still limited, such mechanisms necessitate further investigation. Combining knowledge from previous studies, a minimum level of *β*-glucan, ranging from 4 to 6 g, appears to be essential for its gastrointestinal appetite-regulating effects [321]. However, additional studies addressing the role of dose, form, molecular weight and carrier food on the interaction between *β*-glucan and satiety are still needed before drawing solid conclusions. Moreover, the role of *β*-glucan in long-term weight regulation is still not well understood and needs to be further explored. Inconsistencies in data regarding the effect of dietary or supplementary *β*-glucan on body weight highlight the need for additional research.

## 4. Beta Glucan-Fortified Foods in the Market

### 4.1. Global Dietary Fiber Intake

Insufficient intake of dietary fiber has been reported worldwide. However, the estimates of fiber intake are highly variable.

 In the United States, dietary fiber intake was calculated to be 17 g for males and 12.8 g for females based on the NHANES III study [322]. Based on the results of the Nationwide Food Consumption Survey, a mean dietary fiber intake of 11.4 g per day was reported [323]. Similarly, a mean daily fiber intake of 13.7 g in total, comprising 4.2 g of water-soluble fiber and 6.8 g of water-insoluble fiber, was described based on the Multiple Risk Factor Intervention Trial [324]. In contrast, Hallfrisch et al. [325] and Hermann et al. [326] reported higher intake values, averaging 15 g/day and 18.3 g/day, respectively. Regardless, intakes of dietary fibers in the American population are below levels recommended by the Institute of Medicine (38 g for males and 25 g for females).

In Canada, low daily dietary fiber intakes have been also noted. According to Nova Scotia Department of Health [327], the mean dietary fiber intake was estimated to be 13.5 g per day, ranging from 9.6 g (young women) to 17 g (elderly men). The main sources (88%) of fiber in the diet were reported to be pasta, rice, cereals and breads, vegetables, fruits, and fruit juices [327]. Similarly, in a more recent study on healthy Canadian adolescent males, a median dietary fiber intake of 13.1 g per day was observed [328].

 In Europe, the estimated national values for dietary fiber intake were found to fall within a narrower range: 16 g/day in France [329], 22.1 g/day in Sweden [330], 16.7–20.1 g/day in Finland [331], 21 g/day in Germany [332], and 20–22 g/day in the Netherlands [333]. An exceptionally high intake level of fiber was found in Switzerland, 30–33 g/day, reflecting a positive trend in the eating habits of this population [334]. In the United Kingdom, lower values of 14–16 g/day for men and 18-19 g/day for women were reported [335].

Thus, fiber intakes worldwide are well below the recommended levels despite the recommendations of several health organizations to increase the consumption of foods with high fiber content.

### 4.2. Beta Glucan in Functional Foods

The introduction of fiber into traditional and processed foods provides one method by which to increase fiber intake [81]. Based on consumers' demands for healthier options, the food industry has aimed at developing new products towards functional foods and ingredients.

The best-known examples of functional foods are fermented milks and yoghurts. Several fiber-fortified dairy products are now appearing in market, with inulin being a popular fiber source for such products due to its combined nutritional and technological characteristics [336–341].

Beta glucan is commonly used as a functional ingredient in foods as it is readily available as a byproduct of oat and barley milling and it also provides physiological benefits that are supported by health claims in many jurisdictions. This polysaccharide is also used as a food ingredient in the form of hydrocolloids [342, 343] or as powder using microparticulation [344]. The addition of *β*-glucan into various products, such as baking products, muffins, cakes, pasta, noodles, muesli cereals, milk products, soups, salad dressings, beverages, and reduced-fat dairy and meat products, was found to affect their attributes, including bread making performance, water binding and emulsion stabilizing capacity, thickening ability, texture and appearance, in a concentration-, molecular weight-, and structure-dependent manner [22, 345, 346]. Besides enhancing the nutritional value, *β*-glucans can improve the sensory and gustatory properties of some products. However, the stability of the physiological properties of *β*-glucan when extracted and added to foods has received little examination, leaving uncertain the health benefits of *β*-glucan when incorporated into foods.

In the following sections, the chemical and physiologic functionality of *β*-glucans in food preparations is discussed.

#### 4.2.1. Breakfast Cereals

Oats have been frequently used as an additive in the preparation of cereal products, decreasing water activity and subsequently prolonging durability [81]. Several oat-based breakfast cereals have experienced great success in the market. Adding 20% oat *β*-glucan into chocolate breakfast flakes protected the viability and stabilized the cells of *lactobacillus rhamnosus*, a gut-friendly probiotic bacteria, at temperatures higher than 20°C [347]. As breakfast cereals are commonly consumed in North America, several oat-based hot and cold breakfast cereals are available in the market, making use of *β*-glucan's approved health claims. These products are readily accepted by consumers.

#### 4.2.2. Baking Products

The incorporation of oats into baking products, such as bread, baked goods, and dough, has been widely tested [81]. The incorporation of *β*-glucans to baking products seems promising, ameliorating both sensory characteristics and health properties of products at a maximum concentration of 20%. When oat flour has been substituted for 10% of fine wheat flour in bread, product quality improved in terms of crust color, bread softness, and taste [348]. Moreover, a positive effect of oat *β*-glucan on the sensorial characteristics of biscuits has been described [343]. The addition of the hydrocolloids Nutrim O-B (10% *β*-glucan) and C-Trim-20 (20% *β*-glucan) increased the taste, moisture, and adhesiveness of the product. Similarly, an oat component called Nutrim-5, a hydrocolloid preparation of *β*-glucans produced by treating oat grain or flour with a thermal process, improved the overall strength of pasta without negatively affecting either the quality or the sensory properties [349].

#### 4.2.3. Milk Products

Oats are also used as additives in the production of yogurts with increased amount of fiber [81]. Fiber addition increased the solidity ratio and texture of unsweetened yogurts, accelerated their acidification rate, and increased their viscosity [350]. When substituting fat with *β*-glucans hydrocolloid component at 3.47% and 6.8% in low-fat cheddar cheeses, a softer texture was described with decreased melting time and lowered sensory properties [351]. The addition of oat *β*-glucans concentrate, at 0.7% and 1.4% w/w, to white low-fat cheese products in salt brine improved product texture, while unfavorably affecting its appearance, taste, and odor when compared with the control samples [352]. The probiotic effect of *β*-glucans has been also studied. Beta glucans selectively support the growth of *Lactobacilli *and *Bifidobacteria,* both of them being antagonists to pathogenic bacteria in the digestive system [12, 173]. The addition of oat *β*-glucans to probiotic milk-based drinks, at doses of 0.31–0.36%, increased their stability along with their health benefits [353].

The effects of *β*-glucan on milk sensorial properties have been reported, but results are variable [56, 121, 245, 354]. Oat milk containing *β*-glucan (0.5 g/100 g) was well perceived and got similar sensory evaluation as the control drink (<0.02 g *β*-glucan/100 g) [56]. Sensory evaluations were higher for the milk beverage (500 mL) enriched with 5 g as compared to the one enriched with 10 g of oat and barley *β*-glucan [121]. However, milk enriched with 5 g *β*-glucan had similar sensorial characteristics to the control drink.

In conclusion, the addition of *β*-glucans to yogurts seems to impair their sensory qualities despite improving other rheological properties, irrespective of the dose. On the other hand, addition of *β*-glucans to milk, at doses not exceeding 1%, may provide health benefits without compromising sensorial attributes.

#### 4.2.4. Meat Products

Due to its ability to mimic fat characteristics, oat fiber is one of the most effective ingredients in making low-fat meat products. It can be used to offset the poor quality associated with low-fat beef burgers [355] as well as low-fat sausages [356]. There is no specific study investigating the effect of *β*-glucan, as a fat replacer, on the sensorial attributes and rheological properties of meat products. Thus, future studies should address this applicability option of *β*-glucan.

In conclusion, the introduction of *β*-glucans into food preparations has both beneficial and deleterious impacts. Such impacts mainly depend on the food product to which *β*-glucan is added, in addition to the source, the form, and the dose of *β*-glucan in use. Alterations in the sensorial properties and physiochemical attributes induced by *β*-glucan may be desirable for some products while being detrimental for others.

### 4.3. Challenges of Beta Glucan Fortification

One of the major challenges faced by the functional food industry is developing functional foods with an acceptable taste to the average consumer [357]. Incorporating significant quantities of fiber into food products constitutes a technological challenge due to the possible deleterious effects on textural quality. The addition of fibers may contribute to modifications in the texture, sensory characteristics, and shelf-life of foods due to their water-binding capacity, gel-forming ability, fat mimetic, antisticking, anticlumping, texturising, and thickening effects [358, 359].

Adding *β*-glucan into milk and dairy products was reported to be problematic; first due to its viscosity that may alter the sensory characteristic of foods and second due to its typical slimy texture in the mouth [100]. However, the acceptance rate does not seem to be influenced by the amount of *β*-glucan added to test products but rather by the duration of consumption of these products. Blackcurrant flavored oat milk (0.5 g *β*-glucan/100 g) was well liked among volunteers without differencing it from its counterpart, a rice beverage with the same flavor (<0.02 g *β*-glucan/100 g), at a single evaluation [56]. In addition, the sensory quality of a flavored oat-based fermented product (containing 0.6% *β*-glucan) was acceptable, in comparison to flavored commercial yogurt or nondairy products, in one single taste test [354]. In contrast, when consumed over 5 weeks, oat-based fermented dairy products (0.5-0.6% *β*-glucan) were less preferred than fermented dairy-based control products (<0.05% *β*-glucan) [245]. Similarly, after a period of 5 weeks, beverages with 10 g of barley or oat *β*-glucan were rated lower than those with 5 g of barley or oat *β*-glucan [121]. These findings reflect that, when chronically consumed, *β*-glucan may impair the sensorial perceptions of foods.

Thus, the development of *β*-glucan-fortified foods remains highly challenging as consumers are not willing to accept greater health benefits on the expense of deteriorations in the sensory characteristics of food products.

### 4.4. Effects of Food Processing on the Biological Activities of *β*-Glucan

Food processing alters the physical, chemical, and physiologic characteristics of dietary fibers. Several processing techniques, including cooking, freezing, and storing, affect the physicochemical characteristics of *β*-glucan. Both molecular weight and extractability are important components of the physiological activity of *β*-glucan and both can be affected by food processing [360]. The molecular weight of *β*-glucan in processed oat foods can be smaller than unprocessed. Solubility, which is related to extractability, typically increases initially with processing as depolymerisation occurs and *β*-glucan is released from the cell wall; however, as this degradation continues, solubility decreases as insoluble *β*-glucan aggregates are formed [361]. In products such as oat porridge and oat granola, there is little effect of processing on *β*-glucan molecular weight [172, 362]. However, the molecular weight of *β*-glucan in products such as oat crisp bread decreases by 92% compared to its original oat source [362]. Other studies have also seen reductions in molecular weight in similar products made from different grains [168, 172, 363] and attributed these reductions in molecular weight to the effects of *β*-glucanase enzymes in wheat flour used to make these products [168, 172, 364–366]. These reductions in molecular weight increase with the mixing and fermentation time of the dough [172]. Freezing was also found to affect *β*-glucan solubility. Frozen storage of oat bran muffins significantly lowered *β*-glucan solubility over time, using *in vitro* extraction simulating human digestion [231]. In addition, freeze-thaw cycle reduced the solubility of *β*-glucan in oat bran muffins by 9% to 55% of the fresh values. 

Whether such physicochemical alterations induced by food processing have a significant impact on the established health properties of *β*-glucan is not clear. Effectiveness of *β*-glucan in modulating glucose and insulin parameters is related to dose and viscosity, which can be altered during processing [74]. In fact, 85% of the variation in blood glucose concentrations is explained by the amount of *β*-glucan solubilized and not the total amount originally added to food [367]. On the other hand, the role of viscosity, molecular weight, and solubility, susceptible to modifications by food processing, in regulating *β*-glucan's effect on cholesterol metabolism has not been demonstrated and requires further investigation [74]. 

Thus, since physiologic effects of *β*-glucans may be altered by food processing, it is important to develop a further understanding of such an interaction.

## 5. Summary and Conclusion

It is clear that *β*-glucan is an important food component in the modulation of metabolic dysregulations associated with the metabolic syndrome. However, dose, form, molecular weight, and the carrier food of *β*-glucan shape its effect. The physiological effects of *β*-glucan are mainly attributed to its physicochemical and structural characteristics interacting with the gastrointestinal tract, as reflected by its ability to generate viscous solutions at low concentrations in the upper part of the gastrointestinal tract and to undergo fermentation in the colon.

Although the physiological effects of ingested *β*-glucan are similar to other soluble fibers, its availability and ease of handling leads it to be increasingly incorporated into foods with the purpose of increasing daily fiber consumption. However, challenges in incorporating *β*-glucan into some food items without compromising their sensorial properties and their acceptance by consumers do still exist, and need to be resolved.

##  Conflict of Interests

D. El Khoury, C. Cuda, B. L. Luhovyy, and G. H. Anderson declare that there is no conflict of interests.

## Figures and Tables

**Table 1:** Categorization of recent definitions of fiber based on whether or not a distinction in dietary fiber source is made.

Plant source only
*Food and Agriculture Organization (FAO)/World Health Organization (WHO): *
“Dietary fibre consists of intrinsic plant cell wall polysaccharides” [40]

Categorize fiber types based on source

*Institute of Medicine (IOM): *
“Dietary fiber consists of nondigestible carbohydrates and lignin that are intrinsic and intact in plants
Functional fiber consists of isolated, nondigestible carbohydrates that have beneficial physiological effects in humans
Total fibre is the sum of dietary fibre and functional fiber” [368]
*Health Canada^1^*:
“Dietary fibre consists of the endogenous components of plant material in the diet which are resistant to digestion by enzymes produced by
humans. They are predominantly nonstarch polysaccharides and lignin and may include, in addition, associated substances” [369]
“Novel Fibre or Novel Fibre Source means a food that is manufactured to be a source of dietary fibre, and
(i) that has not traditionally been used for human consumption to any significant extent, or
(ii) that has been chemically processed, for example, oxidized, or physically processed, for example, very finely ground, so as to modify
the properties of the fibre contained therein, or
(iii) that has been highly concentrated from its plant source” [370]
*Codex Alimentarius Commission^2^*:
“Dietary fibre means carbohydrate polymers with ten or more monomeric units, which are not hydrolysed by the endogenous enzymes in
the small intestine of humans and belong to the following categories:
(i) edible carbohydrate polymers naturally occurring in the food as consumed,
(ii) carbohydrate polymers which have been obtained from food raw material by physical, enzymatic, or chemical means and which
have been shown to have a physiological effect of benefit to health as demonstrated by generally accepted scientific evidence to
competent authorities,
Synthetic carbohydrates polymers which have been shown to have a physiological effect of benefit to health as
demonstrated by generally accepted scientific evidence to competent authorities” [30]

No categorization of fibers based on source

*European Food Safety Authority (EFSA): *
“Nondigestible carbohydrates plus lignin” [371]
* Food Standards Australia New Zealand (FSANZ), formerly Australia New Zealand Food Authority (ANZFA)*
“Dietary fibre means the fraction of the edible part of plants or their extracts, or synthetic analogues that
(a) are resistant to the digestion and absorption in the small intestine, usually with complete or partial fermentation in the large
intestine;
(b) promote one or more of the following beneficial physiological effects:
(i) laxation,
(ii) reduction in blood cholesterol,
(iii) modulation of blood glucose,
and includes polysaccharides, oligosaccharides (DP < 2), and lignin” [372]

^1^Health Canada is currently reviewing its definition for fiber and proposed a new definition in December 2010 which has not yet been accepted [373].

^2^Two footnotes have been included with this definition, the first indicates that substances associated with fibre (e.g., lignin, waxes, saponins, etc.) are included in this definition, unless they are isolated and reintroduced into a food. The second footnote states that the decision on whether to include carbohydrates from 3 to 9 monomeric units is up to the discretion of national authorities.
